# Health-seeking behaviours of older black women living with non-communicable diseases in an urban township in South Africa

**DOI:** 10.1186/s12906-016-1378-4

**Published:** 2016-10-24

**Authors:** O. M. Aboyade, R. Beauclair, O. N. Mbamalu, T. R. Puoane, G. D. Hughes

**Affiliations:** 1 South African Herbal Science and Medicine Institute, University of the Western Cape, Bellville, South Africa; 2 The South African Department of Science and Technology/National Research Foundation (DST/NRF) Centre of Excellence in Epidemiological Modelling and Analysis (SACEMA), Stellenbosch University, Stellenbosch, South Africa; 3 International Centre for Reproductive Health (ICRH), Ghent University, Ghent, Belgium; 4 School of Pharmacy, University of the Western Cape, Bellville, 7535 South Africa; 5 School of Public Health, University of the Western Cape, Bellville, South Africa; 6 Present Address: Department of Pharmaceutical Sciences, Tshwane University of Technology, Arcadia, Pretoria, South Africa

**Keywords:** Traditional herbal medicine, Non-communicable diseases, Medical pluralism, Older women

## Abstract

**Background:**

Various studies have shown that non-communicable diseases (NCDs) especially diabetes and hypertension are prevalent among older women living in South African urban areas, placing a heavy burden on the healthcare system. This study aimed to understand the health-seeking behaviour, healthcare practices and prevalence of traditional herbal medicine (THM) use among older women self-reporting NCDs from the Prospective Urban Rural Epidemiology study (PURE).

**Method:**

A homogenous purposive sampling of PURE participants was used to recruit women who were 50 years or older (*n* = 250). Descriptive statistics were used to examine the number of NCDs reported by the study sample, health seeking behaviour and practices as well as THM use. Logistic regression was also employed to investigate possible associations between reported conditions and THM use or medical pluralism.

**Results:**

Within the study sample, 72 % self-reported an NCD. Of those with self-reported NCDs, 46 % had one, and 54 % had two or more NCDs. Those with NCDs usually visited public clinics (80 %), relied on doctors (90 %) and nurses (85 %) for health information, and mostly used conventional medicine (CM) to manage high blood pressure (81 %). About 30 % of those with NCDs indicated using THM, of whom 29 (53 %) reported practicing medical pluralism. Participants with dental problems (OR: 3.24, 95 % CI: 1.30–8.20), headaches (OR: 2.42, 95 % CI: 1.24–4.94), heart burn (OR: 2.30, 95 % CI: 1.18–4.48) and severe tiredness (OR: 2.05, 95 % CI: 1.08–3.99) were more likely to use THM. Anxiety and allergies increased the likelihood to practise medical pluralism by five and 20 times, respectively.

**Conclusion:**

Self-reported NCD with co-morbidities was prevalent among the participants in the study. Most of the study participants utilized state-owned clinics and hospitals for the management of their chronic conditions. THM use was not very common. However, among those who used THM, medical pluralism was prevalent. Family history was the most common reason for THM use, with many THM patrons utilizing these for treatment of a health condition. Older black women with anxiety and allergies were more likely to practise medical pluralism.

## Background

Non-communicable diseases (NCDs), accounting for about two-thirds of deaths globally (35 million), pose a significant public health challenge. More than 80 % of these deaths occur in low- and middle-income countries, with two-thirds of such deaths in people older than 60 years [1]. With increasing age, people are more susceptible to developing at least one NCD, with the attendant co-morbidities, and are also at higher risk of long-term consequences of NCDs [2]. According to the NCD Alliance [3], NCDs are the leading cause of death in women, especially in Africa. This has been corroborated by studies that reported being female, older than 50 years, and living in urban cities in sub-Saharan Africa as predictors of NCDs [4–6].

In sub-Saharan Africa, the epidemiological transition has seen a shift in mortality from mostly infectious diseases to chronic NCDs [7]. Life expectancy in South Africa has been gradually increasing in recent years. Statistics for 2015, indicated life expectancy in the country at 60 and 64 years old for males and females respectively [8]. Presently in South Africa, there are about 4.42 million people older than 60 years, representing 8 % of the total population [8]. The aging population is expected to further burden the already challenged healthcare system. This is of great concern given the high prevalence of NCDs and their attendant morbidity and mortality. Many studies have reported a prevalence exceeding 65 % for chronic diseases among older people (≥65 years) [9]. A similarly high prevalence has also been reported in sub-Saharan African countries [10]. Age-standardized death rates from NCDs are reportedly higher in some sub-Saharan African countries (Democratic Republic of the Congo, Nigeria, Ethiopia and South Africa) than in high income countries, with older women more at risk than older men [11]. Such risk of NCD may increase with urbanization and its attendant lifestyle modifications, especially in developing countries.

In north India, older members of the community mostly sought healthcare at government institutions (> 80 %) while the rest preferred ayurvedic or homeopathic methods [12]. Moreover, the study revealed that urban dwellers were more likely to seek healthcare than their rural counterparts [12]. In South Africa, older people in urban areas rated their health poorer than their counterparts in rural areas [13]. In the afore-mentioned study, over 80 % of respondents in urban and rural areas utilized conventional healthcare institutions or practitioners, with less than 10 % utilizing the services of a traditional healer [13]. Within South Africa, older women play major roles in the social, cultural and economic spheres of their families and communities. This is especially so in the wake of the HIV/AIDS pandemic experienced over the last two decades [14–16], which has claimed the lives of many middle-aged adults. Many older women report lower health status, higher disability, and poorer quality of life compared to their male counterparts [17]. This situation is not peculiar to South Africa; Aboderin [18], also reported that access to healthcare and essential medicines for NCDs is a challenge for the elderly in Africa. Such access to healthcare may be limited for older women in light of their multi-tasking roles and perhaps, cultural restrictions. Lack of income and limited transportation to hospitals within the continent are additional barriers which may be faced by elderly people with NCDs in their quest for proper healthcare [18, 19]. We hypothesize that these challenges may prompt older women in a South African peri-urban/township area to seek readily accessible treatment options to take care of their health conditions. Although studies on health-seeking behaviour of older women in Australia and America exists, there are few indicating how older women’s healthcare needs in Africa are met [20, 21]. This study, therefore, aims to explore the health-seeking behaviour, healthcare practices and prevalence of traditional herbal medicine (THM) use among a sub-sample of elderly female participants from the Prospective Urban Rural Epidemiology (PURE) study —South Africa, who self-reported having NCDs.

## Methods

### Study design and setting

A cross-sectional descriptive study was conducted by taking a sample of participants from the South African arm of a large prospective study – the PURE study. This study comprises a global cohort investigating the impact of socio-environmental transitions on the health of over 150,000 adults. The recruited adults were initially between ages 35 and 75 years old, from communities in 17 low-, middle- and high-income countries. A detailed description of the PURE study design has been published by Teo et al. [22]. The present study was conducted in Langa, which is one of the oldest and poorest [23] black South African townships, in Cape Town, South Africa. This township was initially established for the high population of migrant black South Africans, who settled here because of its lower living costs, proximity to the city, and available transport resources [24]. Unemployment in Langa is high and the education level is generally lower than that of high (secondary) school [25]. Most of the respondents in this study were children of internal migrants.

### Population and sampling

Sampling for this study was a homogenous purposive sampling. The current study recruited all women who were 50 years and older (*n* = 250) from the participants of the PURE THM study (*n* = 456), who in turn were purposively recruited from the Langa (a peri-urban community) PURE cohort (*n* = 1000). Socio-demographic details of the PURE THM study population as well as a breakdown of these details by their traditional herbal medicine use, have been reported elsewhere [26].

### Definitions

Conventional medicine (CM) in this study is defined as medicine prescribed by clinicians. Traditional herbal medicine is defined as herbs or herbal products that contain plant parts or plant materials or their combinations as active ingredients. Complementary and alternative medicines (CAM) are groups of distinct health practices either used in combination with or in place of conventional medicines. Medical pluralism is defined as the use of different treatment approaches for healthcare. In the context of this study, medical pluralism refers to the utilization of both CM and THM for healthcare or disease management. While this term is usually applied to healthcare systems, it is used to describe individual choices with respect to healthcare approaches in the study context.

### Data collection

Data collection methods have been reported in detail by Hughes et al. [26]. Using the participants’ residential addresses obtained in the PURE study, trained interviewers visited the households/individuals between October 2013 and August 2014. Structured questionnaires were administered through face-to-face interviews. The questionnaire was developed using the following questionnaires as guidelines:

1. Australian Longitudinal study on Women’s health, 6^th^ survey for the women of 1946–51 cohort, 2010.
2. Australian Longitudinal study on Women’s health, Alternative medicine use among women of 1946–51 cohort, 2009.
3. PURE Study questionnaire (South Africa) [22].
4. Assessment of migrant populations’ needs and vulnerabilities in Gauteng province, South Africa, 2012 survey.

The structured questionnaire was pre-tested and revised in 2012. Thereafter, a pilot study was conducted before the questionnaire was used for the study. The interviews were conducted by five trained data collectors in isiXhosa, the language of the residents in that community. Data were collected on the respondents’ self-reported demographic characteristics (age, sex, education, marital and employment status), clinical/medical history, health-seeking behaviour and THM use (duration of use, condition for use, dosage, and form). The quality of data collected was maintained through the use of standardized protocols and centralized training.

### Statistical analysis

The statistical analysis was conducted using R statistical programming, version 3.1.1. [27]. Using frequency distributions, relationships between socio-demographic variables and the number of self-reported NCDs in women who were 50 years or older at the time of the study were explored. Women were defined as having an NCD if they self-reported a clinical diagnosis of hypertension, diabetes, arthritis, cancer, heart or cardiovascular disease, stroke, depression, hypercholesterolemia, and/or asthma. They were then categorized into groups based on self-reporting ‘None’, ‘One’, or ‘Two or more’ conditions.

Frequency distributions were calculated for different health behaviours reported by these women, who had reported an NCD. Of primary interest to this study was the types of health facilities visited by the study population; the means of accessing health information; types of CM used; and use of THM. The characteristics of THM use were also assessed among women who were 50 years or older, with NCD, and who reportedly use THM. In this same sub-sample, the prevalence of THM use for different self-reported NCDs was also graphically examined.

Finally, the relationship between different reported conditions and use of only THM or THM in combination with CM (medical pluralism), among older women who self-reported having an NCD, was examined. For this analysis, logistic regression was employed to calculate crude odds ratios (ORs) and 95 % confidence intervals (95 % CIs). The relationship between participants’ NCD co-morbidities and odds of using THM or medical pluralism were also examined. Women were considered to have a co-morbid NCD if they reported two or more diagnoses of the mentioned NCDs. In this analysis, the primary focus was on predictors of THM use and medical pluralism using marginal associations, rather than building causal models that adjust for potential confounders.

All tabulations included missing data. The missing data were primarily a result of data capture error and were assumed to be missing completely at random—in a way that is not related to the variables of interest. In addition, for any given variable studied in the sub-population of interest, less than 10 % of participants had missing data. When jointly considered, the missing values are believed not to result in undue bias of results.

## Results

Among the 250 women in the study sample, 72.4 % (*n* = 181) had at least one NCD, while 27.6 % (*n* = 69) did not report any NCD diagnosis. Of the 181 who reported having NCDs, 46.1 % (*n* = 83) reported having one NCD, while 53.9 % (*n* = 98) reported two or more NCDs. Table 1 provides a description of the socio-demographic characteristics among the population for different numbers of NCD diagnoses. In all categories, most of the women were unmarried, with secondary education, unemployed, primarily of Christian religion, and resided in the place they were born or from where they originated. While the general trends look similar, a greater proportion of women who had two or more NCDs were unemployed: 98.1 % versus 79.7 and 83.1 % for no NCDs and one NCD, respectively. Likewise, a greater proportion of women with co-morbid NCDs were widowed, divorced, or separated compared to women with no or one NCD (35.7 % versus 20.3 and 30.1 %, respectively).

**Table 1:** Relationship between the number of non-communicable diseases (NCDs) self-reported and socio-demographic variables for older women (*n*=250)

Socio-demographic characteristics	Self-reported NCDs Number (%)		
	0	1	≥2
Prevalence of number of NCDs	69 (27.6)	83 (33.2)	98 (39.2)
Marital status			
Never married	39 (56.5)	40 (48.2)	39 (39.8)
Married/cohabiting	15 (21.7)	17 (20.5)	21 (21.4)
Widowed, divorced, separated	14 (20.3)	25 (30.1)	35 (35.7)
Missing	1 (1.4)	1 (1.2)	3 (3.1)
Highest education level			
None or primary	23 (33.3)	27 (32.5)	30 (30.6)
Secondary (8−12)	42 (60.9)	47 (56.6)	62 (63.3)
Tertiary or other	4 (5.8)	8 (9.6)	5 (5.1)
Missing	0 (0.0)	1 (1.2)	1 (1.0)
Employment			
No	55 (79.7)	69 (83.1)	90 (91.8)
Yes	10 (14.5)	10 (12.0)	6 (6.1)
Missing	4 (5.8)	4 (4.8)	2 (2.0)
Monthly household income			
<R2000 PM	59 (85.5)	65 (79.3)	82 (83.7)
R2000−R5000 PM	9 (13.0)	14 (16.9)	13 (13.3)
R5000−R10000 PM	0 (0.0)	4 (4.8)	2 (2.0)
Missing	1 (1.4)	0 (0.0)	1 (1.0)
Religion			
Christian	66 (95.7)	80 (96.4)	95 (96.9)
Other	1 (1.4)	2 (2.4)	2 (2.0)
Missing	2 (2.9)	1 (1.2)	1 (1.0)
Medical aid			
No	63 (91.3)	74 (89.2)	92 (93.9)
Yes	5 (7.2)	5 (6.0)	3 (3.1)
Missing	1 (1.4)	4 (4.8)	3 (3.1)
Place of origin=place of birth			
No	21 (30.4)	28 (33.7)	35 (35.7)
Yes	47 (68.1)	54 (65.1)	63 (64.3)
Missing	1 (1.4)	1 (1.2)	0 (0.0)

Among the women with NCDs, 79.6 % used local public clinics, while the use of (Table 2) district public hospitals was also relatively common, with 39.8 % reportedly visiting these. None of the participants indicated consultation of a traditional practitioner for their healthcare. The most common sources of health information for the sub-population of participants were doctors (89.5 %), nurses (85.1 %), radio (54.1 %) and the television (53.0 %). The most frequently used classes of CM at the time of the study were anti-hypertensives (80.7 %), pain relievers (43.1 %), anti-hyperlipidaemic agents (23.8 %), and diuretics (23.2 %). Moreover, 55 (30.5 %) of the older women with NCDs were also using THM.

**Table 2:** Health behaviours of older women with non-communicable diseases (*n*=181)

Variable	Number (%)
Access to healthcare:	
Local public clinic	144 (79.6)
Private clinic	6 (3.3)
District public hospital	72 (39.8)
Private hospital	3 (1.7)
General hospital	35 (19.3)
Mobile clinic	1 (0.6)
Traditional practitioner	0 (0.0)
No one, self-medicates	1 (0.6)
Receives health information:	
Doctor	162 (89.5)
Nurse	154 (85.1)
Other health professionals	31 (17.1)
Programmes/organizations	36 (19.9)
Books	50 (27.6)
Internet	10 (5.5)
TV	96 (53.0)
Radio	98 (54.1)
Family	85 (47.0)
Private health funds	9 (5.0)
Classes of conventional medicines (CM) used/conditions treated with CM:	
High blood pressure	146 (80.7)
Pain	78 (43.1)
Anti-retrovirals	5 (2.8)
Heart	10 (5.5)
Diuretics	42 (23.2)
Allergies	7 (3.9)
Diabetes	41 (22.7)
Anti-coagulants	40 (22.1)
Dietary supplements	15 (8.3)
Anti-depressants	16 (8.8)
Epilepsy	1 (0.6)
Anti-inflammatory agents	13 (7.2)
Ulcers	3 (1.7)
Antibiotics	1 (0.6)
Arthritis	6 (3.3)
Muscle relaxants	1 (0.6)
Asthma	16 (8.8)
Cholesterol	43 (23.8)
Reported traditional herbal medicine use	55 (30.4)

**Fig. 1:**
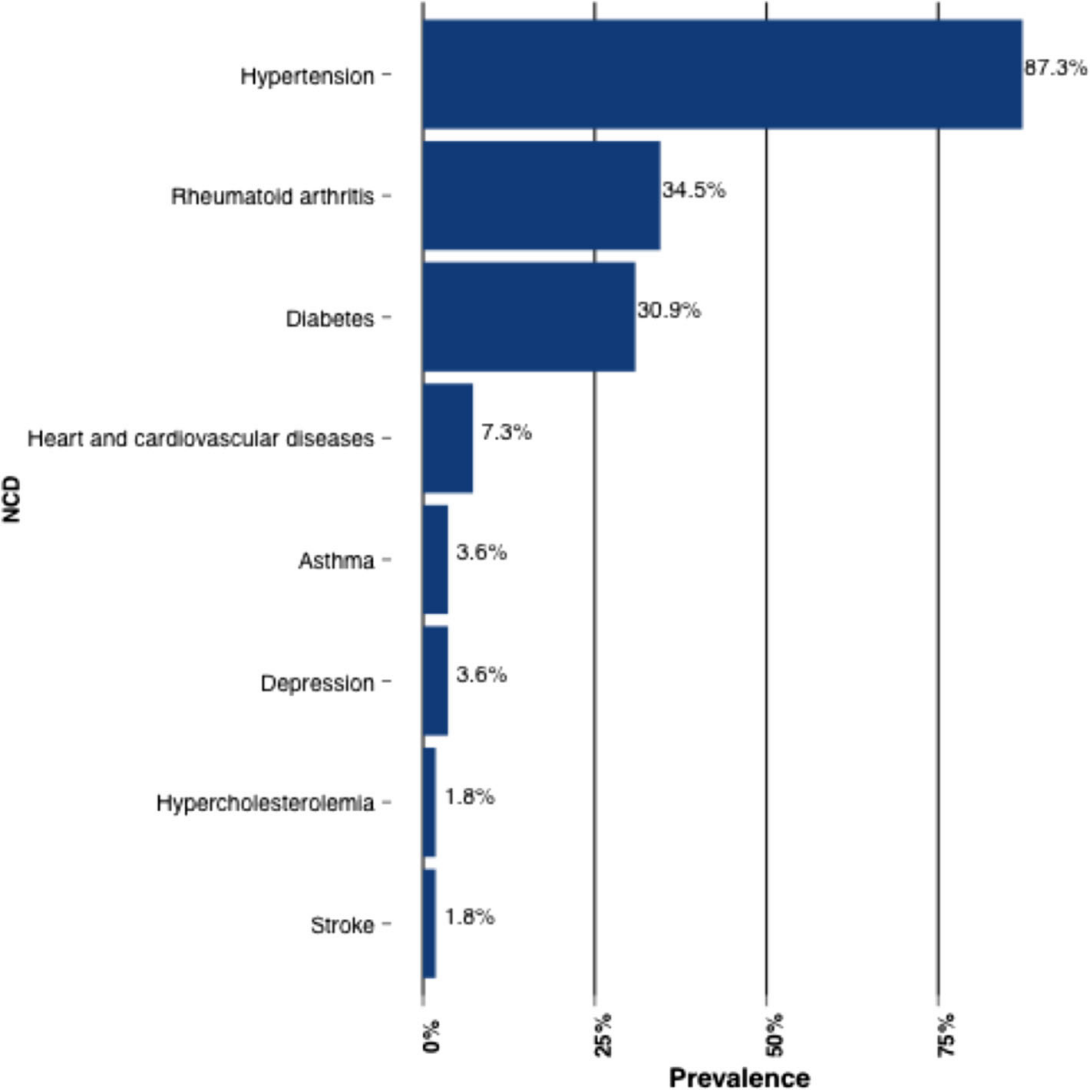
Prevalent non-communicable diseases among older women who use traditional herbal medicine (*n* = 55). (There were no cases of self-reported cancer in this sub-population, so this was eliminated from the figure.)

In Table 3, this subset of women is further examined and characterized according to their THM use. The most cited reasons for using THM were family history of use (47.3 %), treating conditions (45.5 %), positive recommendations from others (34.5 %), and cultural beliefs (21.8 %). Many of these women obtained THM from markets (43.6 %), and most of them (80.0 %) use THM in the form of a tea. Only seven (12.7 %) participants disclosed their use of THM to their healthcare practitioners, even though 41.8 % said they used THM ‘often’ or ‘always’ and in combination with CM (52.7 %). Figure 1 shows that 81.3 % of these women reported being hypertensive. Rheumatoid arthritis (34.5 %) and diabetes (30.9 %) were other NCDs also frequently reported among these women.

**Table 3:** Characteristics of traditional herbal medicine (THM) use among older women with non-communicable diseases (*n*=55)

Variable	Number (%)
Reason for use:	
Family history	26 (47.3)
Cultural beliefs	12 (21.8)
Low cost	9 (16.4)
Easily accessible	7 (12.7)
Positive recommendation	19 (34.5)
Conventional medicine failure	0 (0.0)
Cures diseases	3 (5.5)
Treats side effects	3 (5.5)
Doctor recommended	1 (1.8)
Treat a condition	25 (45.5)
THM obtained:	
Market	24 (43.6)
Traditional practitioner	9 (16.4)
Personal harvest	11 (20.0)
Pharmacy	11 (20.0)
Over the counter	3 (5.5)
THM Administration:	
Tea	44 (80.0)
Decoction	4 (7.3)
Powder	4 (7.3)
Extract	8 (14.5)
Tablet	1 (1.8)
Topical	1 (1.8)
Discloses use of conventional medicine to traditional practitioner	6 (10.9)
Discloses use of THM to health care practitioner	7 (12.7)
Uses THM ‘often’ or ‘always’	23 (41.8)
Uses THM in combination with conventional medicine (medical pluralism)	29 (52.7)

Various reported conditions were examined to see if they increased a participant’s odds of using THM or THM in combination with CM, among the older women with NCDs (Table 4). No evidence could be provided for co-morbidities increasing a participant’s odds of using THM or practising medical pluralism. However, a history of “heart burn” (OR 2.30, 95 % CI: 1.18–4.48); headaches (OR 2.42, 95 % CI: 1.24–4.94); severe tiredness (OR 2.05, 95 % CI: 1.08–3.99); dental problems (OR 3.24, 95 %: 1.30–8.20); dizziness or loss of balance (OR 1.94, 95 % CI: 1.00–3.75); and heart palpitations (OR 4.57, 95 % CI: 1.79–12.27) all increased the participants’ likelihood of using THM. Those who reported having allergies had almost 20-fold increased odds of using THM in combination with CM (95 % CI: 4.62–138.10) compared to those who did not report allergies. Study participants reporting anxiety also had five times the odds (95 % CI: 1.22–38.19) of medical pluralism compared to those who did not report anxiety.

**Table 4:** Odds of using only traditional herbal medicine (THM) and THM in combination with conventional medications for different reported health conditions in older women with non-communicable diseases (NCDs)

	THM use		Medical pluralism^a^	
	Number (%)	Crude OR (95 % CI)	Number (%)	Crude OR (95 % CI)
Comorbidity with another NCD	31 (56.4)	1.14 (0.60–2.16)	17 (58.6)	1.21 (0.42–3.57)
Allergies	20 (36.4)	1.32 (0.67–2.57)	18 (62.1)	19.64 (4.62–138.10)
Breathing difficulties	13 (23.6)	0.80 (0.38–1.65)	8 (27.6)	1.60 (0.46–6.06)
Heart burn	25 (45.5)	2.30 (1.18–4.48)	13 (44.8)	0.95 (0.33–2.76)
Pains in the chest	17 (30.9)	1.16 (0.57–2.31)	6 (20.7)	0.36 (0.10–1.14)
Headaches	40 (72.7)	2.42 (1.24–4.94)	23 (79.3)	2.03 (0.61–7.11)
Severe tiredness	35 (63.6)	2.05 (1.08–3.99)	19 (65.5)	11.19 (0.39–3.61)
Stiff or painful joints	35 (63.6)	1.78 (0.93–3.50)	18 (62.1)	0.95 (0.31–2.93)
Dental problems	12 (21.8)	3.24 (1.30–8.20)	6 (20.7)	0.87 (0.24–3.20)
Bowel problems	10 (18.2)	1.18 (0.49–2.67)	5 (17.2)	0.88 (0.22–3.55)
Dizziness or loss of balance	24 (43.6)	1.94 (1.00–3.75)	12 (41.4)	0.82 (0.28–2.40)
Sadness	15 (27.3)	1.68 (0.79–3.52)	9 (31.0)	1.5 (0.45–5.23)
Back pains	27 (49.1)	1.47 (0.77–2.78)	16 (55.2)	1.68 (0.58–4.98)
Anxiety	11 (20.0)	1.85 (0.77–4.32)	9 (31.0)	5.40 (1.22–38.19)
Heart palpitations	13 (23.6)	4.57 (1.79–12.27)	9 (31.0)	2.48 (0.69–10.31)

^**a**^Those who use conventional medicine in conjunction with THM

## Discussion

The socio-demographic details of the comprehensive PURE THM study population as well as a breakdown of these details by THM use, have been reported elsewhere [26]. The present study explores the health-seeking behaviour, healthcare practices and prevalence of THM use among elderly women with self-reported NCDs, who participated in the South African leg of the PURE study. About three-quarters of the study sample reported having an NCD, with more than half of them self-reporting two or more NCDs.

NCDs have been considered to be poorly managed in South Africa as a result of infrequent access to such healthcare [28]. This is despite the fact that healthcare was rated by participants in the reported study as accessible and affordable, indicating the need for more research aimed at addressing barriers to healthcare utilization [28]. With regard to their health-seeking behaviours, most of the participants in the present study visit public clinics (the community health centres, CHCs), and very few of them utilized the district public hospitals or general hospitals. This is probably because of the fact that the first port of call for patients presenting to the public healthcare sector in South Africa is the local CHC. Generally, patients who utilize the public healthcare system may not present at the district public hospital without referral from the CHC.

Co-morbidities have been found to be a common phenomenon among the elderly [12, 29]. The prevalence of co-morbidities was higher (54 %) in the present study compared to those previously reported for South Africa by Phaswana-Mafuya et al., [4] (22.5 %) and Ibanez-Gonzales and Norris, [28] (19.9 %). This variation in the reported prevalence of NCDs might be attributable to the number of diseases investigated in both previous studies; generally, the higher the number of diseases, the higher the prevalence of co-morbidity [30]. However, studies conducted in other parts of the world such as the United States (65 %) [31] and Bangladesh (53.8 %) [30], reported a prevalence of co-morbidity similar to that of the present study. This present study indicates that participants with co-morbidities were less likely to use THM than those without co-morbidities. This is in contrast to a study conducted in the United States which aimed to examine the association between the type of multimorbidity and complementary and alternative medicine CAM use among adults with multimorbidity [32].

Older women, particularly those within the ages 50 and 64 years, as well as those living in rural areas or non-urban environments are also known to be major users of CAM [33, 34]. In the present study, about a third of the respondents with NCDs used THM. Among older women who had NCDs and used THM, a large fraction reported having hypertension, a finding supported by several other studies [35–37]. Several factors influence THM use among older women. According to McLaughlin et al., [34] personal beliefs and social networks are influences to CAM use. The present study showed that THM use was associated with family history and positive recommendations from other individuals. Interestingly, cultural beliefs were not a very popular reason for THM use among the study participants. The reason for this might be because most of the participants in this study were born in Langa, an urban area. For them, the term ‘cultural beliefs’ may be something associated with a rural lifestyle, with which they have never identified.

The present study also documents that THMs are used extensively to treat health conditions, which differed from other studies where CAM/THM were used more for health maintenance [34, 37, 38]. Symptoms such as heart palpitation, headaches, severe tiredness, and dental problems all had a positive association with THM use (Table 4). According to Alwhaibi et al., [32] the presence of a physical illness with a chronic condition was associated with CAM use in their study. The positive association reported in the present study may perhaps also be by reason of the participant’s view of these symptoms as merely self-limiting, without the need for specialised care. Hence, their preference to self-medicate rather than consult a healthcare practitioner. Medical professionals’ awareness of older women’s habits to treat specific symptoms with THM, will help them in prescribing efficacious medicines to alleviate these symptoms. This will simultaneously reduce adverse effects or drug-herb interactions which may be caused by THM use alone or in combination with CM.

While THMs were used frequently and in combination with CM, most of the respondents did not disclose their use of THM to their healthcare practitioners. This is despite the fact that health information is received by participants mostly from conventional healthcare practitioners. Such non-disclosure of THM use is not peculiar to this study, and has been reported in other studies [37, 39, 40]. However, this is in contrast to findings from studies conducted in Australia [27] and the United States [20], where older women disclosed CAM use to their healthcare providers. Reasons for non-disclosure of THM use are quite diverse and range from fear of discrimination by healthcare workers to lack of required treatments at clinics and hospitals [39, 41].

The present study showed that older women with anxiety are five times more likely to practice medical pluralism. This is supported by findings from another study conducted among patients attending four primary care facilities in the United States, where adults with anxiety and chronic conditions are known to use CAM more than those who do not report anxiety [42]. The high prevalence of THM use among patients with anxiety has been attributed to the holistic nature of the treatment and to patients’ dissatisfaction with CM used for the treatment of this disorder. For instance, some classes of anti-anxiety drugs may worsen anxiety symptoms or result in undesirable side effects, making patients to feel more anxious about their apparent lack of control over their life [43]. In an effort to address these symptoms or side effects, such patients may attempt to regain the desired control by the use of THM, a move which increases the prevalence of THM use among this sub-population [44].

Older women with allergies were also 20 times more likely to use THM in conjunction with CM. This estimate should, however, be interpreted with caution, as the confidence interval is very wide. Common allergies may in some cases be viewed by individuals as ‘harmless’ and merely self-limiting, a condition which participants may then have tried to control through the use of THM as opposed to CM. This particularly may be the case, given the view that complementary treatment measures (such as THM use) are seen to be holistic, i.e., treating the whole person as opposed to only treating symptoms of an ailment [44].

There are several study limitations that should be addressed. First, the use of a small sample of people in a peri-urban disadvantaged community from Cape Town, South Africa, which is primarily composed of one ethnic group. This is because the participants were a sub-set from a larger study. Therefore, the results cannot be generalizable to other contexts within South Africa, let alone other countries. Second, the study utilized self-reported measures of NCDs which were not cross-checked with any medical records or doctor’s diagnoses. Therefore, the chance is that the study could have suffered from misclassification. Since it was not the study objective to report true prevalence of these conditions, these limitations will probably have little effect on result interpretation. In the South African setting, such information is of utmost importance, especially when viewed against the background of older women’s major roles at family and community levels.

### Conclusion and recommendation

Most of the older women in this study self-reported having an NCD, with over half of them having co-morbidities. In terms of health-seeking behaviour, most of the participants utilized state-owned public health facilities. No statistically significant difference in THM use was observed between those with co-morbidities and those without. THM was used mainly to treat a health condition. A few of the older black women with NCDs used THM, with many of them also practicing medical pluralism. Those with anxiety and allergies were also more likely to practise medical pluralism.

Learning how to care for and treat older black South African women, especially those with multiple NCDs, is very important in the public health discourse. This is because of their social and economic relevance within their communities. Awareness around older women with multiple NCDs/co-morbidities and their use of alternative treatment modalities, especially within African cultural paradigm is important. Therefore, healthcare workers should be more observant of medical pluralism, and educate patients on the importance of disclosure of their THM use. Furthermore, healthcare workers should be educated about the importance of questioning older women about THM use during consultations. Enabling conditions to improve the communication gap between older women with NCDs and the health caregivers are also required. This would create awareness and room for education on the potential benefits cum downfalls of possible drug interactions which may arise from medical pluralism, and which may ultimately influence patient therapy.

This study is just the beginning of efforts to understand the health-seeking behaviours among older black South African women. Future studies should also investigate THM use in other contexts.

## Abbreviations

CAM: Complementary and alternative medicine
NCDs: Non-communicable diseases
PURE: Prospective urban and rural epidemiology
THM: Traditional herbal medicine
